# Anti-Adipogenic Activity of High-Phenolic Sorghum Brans in Pre-Adipocytes

**DOI:** 10.3390/nu14071493

**Published:** 2022-04-02

**Authors:** Hee-Seop Lee, Ádina L. Santana, Jaymi Peterson, Umut Yucel, Ramasamy Perumal, Joaquin De Leon, Seong-Ho Lee, Dmitriy Smolensky

**Affiliations:** 1 Department of Nutrition and Food Science, College of Agriculture and Natural Resources, University of Maryland, College Park, MD 20742, USA; heeseopl@uark.edu; 2 Department of Animal Sciences and Industry, Food Science Institute, Kansas State University, Manhattan, KS 66503, USA; adina@ksu.edu (Á.L.S.); lawrencejay@ksu.edu (J.P.); yucel@ksu.edu (U.Y.); 3 Agricultural Research Center, Department of Agronomy, Kansas State University, Hays, KS 67601, USA; perumal@ksu.edu; 4 Grain Quality and Structure Research Unit, United State Department of Agriculture, Agricultural Research Service, Manhattan, KS 66502, USA; joaquin.deleon@usda.gov

**Keywords:** high-phenolic sorghum bran, adipogenesis, lipogenesis, 3T3-L1, polyphenols

## Abstract

Obesity is one of the leading public health problems that can result in life-threatening metabolic and chronic diseases such as cardiovascular diseases, diabetes, and cancer. Sorghum (*Sorghum bicolor* (L.) Moench) is the fifth most important cereal crop in the world and certain genotypes of sorghum have high polyphenol content. PI570481, SC84, and commercially available sumac sorghum are high-polyphenol genotypes that have demonstrated strong anti-cancer activities in previous studies. The objective of this study was to explore a potential anti-obesity use of extracts from sorghum bran in the differentiation of 3T3-L1 preadipocytes and to investigate cellular and molecular responses in differentiated adipocytes to elucidate related mechanisms. None of the four different sorghum bran extracts (PI570481, SC84, Sumac, and white sorghum as a low-polyphenol control) caused cytotoxicity in undifferentiated and differentiated 3T3-L1 cells at doses used in this study. Sorghum bran extracts (PI570481, SC84, and Sumac) reduced intracellular lipid accumulation and expression of adipogenic and lipogenic proteins in a dose-dependent manner in differentiated 3T3-L1 cells. The same polyphenol containing sorghum bran extracts also repressed production of reactive oxygen species (ROS) and MAPK signaling pathways and repressed insulin signaling and glucose uptake in differentiated 3T3-L1 cells. These data propose a potential use of high-phenolic sorghum bran for the prevention of obesity.

## 1. Introduction

The incidence of obesity sharply increased in the 21st century, thereby predisposing the population to a variety of obesity-related diseases, such as type 2 diabetes, cardiovascular disease, and cancer. The significance of plant-based diets in the prevention and management of obesity and metabolic diseases has received increasing attention for the past few decades [1]. Numerous epidemiological studies suggest that including whole grains in the diet reduces the risk of both obesity and weight gain. Moreover, a systematic review of previously published studies found a modest reduction in body mass index (BMI) and waist circumference in people who consumed three servings of whole grains daily [2]. 

Sorghum (*Sorghum bicolor* (L.) Moench) ranks in the fifth place within the most cultivated cereal crops destined for human consumption worldwide. In addition, sorghum is among the most efficient crops in the conversion of both solar energy and water use and is known as a high-energy and drought-tolerant crop that is environmentally friendly [3]. Due to its wide uses and ability to adapt, sorghum is an important crop during the era of climate change. In addition to the abundance of major nutrients (starch, proteins, lipids, minerals, and vitamins), sorghum contains a diverse and extensive distribution of polyphenolic compounds mainly located in the bran. The phenolic compounds include phenolic acids (benzoic and cinnamic acids), flavonoids (anthocyanidins), and tannins (proanthocyanidins and flavin-3-ols) [4,5].

Sorghum-derived phenols were shown to have potential health benefits in the management of obesity and diabetes [6]. Several preclinical studies reported anti-obesity activities of sorghum extracts. Significant weight loss was observed from animals fed sorghum-containing diets. For example, corn-based diets supplemented with tannin-rich sorghum significantly lowered the body weight gain and feed conversion ratio relative to the corn-based control diet without sorghum in rabbits [7]. Consumption of ethyl acetate-based sorghum extract repressed obesity in high-cholesterol-containing-diet-fed hyperlipidemic rats [8] and feeding diets containing extruded sorghum flour showed significantly lower BMI in high-fat-diet-induced obese rats [9]. *Hwanggeumchal* sorghum extract was shown to improve insulin sensitivity in mice fed with a high-fat diet [10]. In addition, another study showed that extruded sorghum decreases adiposity and inflammation in diet-induced obese rats; however, the study used only a single sorghum genotype [11]. Interestingly, a sorghum drink containing high levels of proanthocyanidin (condensed tannins) performed better than the two other sorghum drinks that did not contain proanthocyanidins at reducing blood glucose [12]. Human clinical trials also indicated that consumption of flaked biscuits made from whole grain sorghum led to higher satiety compared to participants receiving the wheat treatment [13] and consumption of extruded sorghum combined with a calorie-restricted diet significantly promoted weight loss and decreased waist circumference and body fat percentage and performed better than wheat [14].

There is still a lack of mechanistic understanding in the anti-obesity activity of sorghum phenolic compounds and its components. In addition, the level of phenolic compound content is affected by several factors including the sorghum genotype, environmental stress, and extraction methods [15]. While such findings are of great value to human health, they lack information regarding the mechanism of function and fail to identify a sorghum genotype(s) with a high amount of relevant bioactive compounds.

Recently, we identified novel high-phenolic sorghum grains (accession number: PI570481 and SC84) that exerted in vitro anti-cancer activity [16,17]. Likewise, the commercial variety Sumac sorghum is considered a high-phenolic genotype that is available to consumers.

The current study was designed to examine if and how phenolic sorghum bran extracts from novel sorghum genotypes PI570481 and SC84, as well as commercially available Sumac and F1000 (white) varieties, affect the differentiation of preadipocytes to adipocytes and fat accumulation in the 3T3-L1 cell line.

## 2. Materials and Methods

### 2.1. Materials

The 3T3-L1 preadipocytes were purchased from American Type Culture Collection (Manassas, VA, USA). Antibodies for C/EBPα (#8178), PPARγ (#2435), FAS (#3180), adiponectin (#2789), perilipin (#9349), p-AKT (#4060), AKT (#4691), Bim (#2933), β-catenin (#9582), pERK (#9101), ERK (#9102), p-p38 (#9211), p38(#9212), p-JNK (#9251), JNK (#9258), pAMPK (#2535), AMPK (#5831), IRS-1 (#3407), PARP (#9542), and β-actin (#5125) were purchased from Cell Signaling (Danvers, MA, USA), Antibodies for Bak (sc-832) was purchased from Santa Cruz Biotechnology (Santa Cruz, CA, USA). All chemicals including cell culture media were purchased from Fisher Scientific (Waltham, MA, USA), unless otherwise specified.

For the HPLC, the solvents (analytical grade) ethanol, methanol, acetonitrile, and glacial acetic acid were obtained from Fisher Scientific (Waltham, MA, USA). The analytical standards of luteolin, luteolinidin, apigenin, kaempferol, catechin, and epicatechin were obtained from Indofine Chemical Company (Hillsborough, NJ, USA); eriodictyol and apigenidin were obtained from Sigma-Aldrich (St. Louis, MO, USA); 7-methoxy apigenidin was obtained from Cayman Chemicals (Ann Harbor, MI, USA); naringenin was obtained from SAFC (Lenexa, KS, USA); the p-coumaric acid, caffeic acid, ferulic acid, vanillic acid, and chlorogenic acid were obtained from MP (Santa Ana, CA, USA).

### 2.2. Sorghum Processing

Sorghum brans used in this study were from four sorghum genotypes: (1) PI570481 (a high-phenolic/high-tannin black grain), (2) SC84/PI534144 (a high-phenolic/high-tannin brown grain) [18], (3) Sumac sorghum (a moderately high-polyphenol/-tannin brown grain that is in commercial use in the United States), and (4) white sorghum (a grain with low phenolic levels, used as a control). PI570481 and SC84 were grown by Kansas State University in Puerto Vallarta, Mexico, during the 2016–2018 winter nursery seasons. Sumac and white sorghum were purchased from Nulife Market (Scott City, KS, USA).

Grain samples were ground using an UDY Mill (UDY Corporation Cyclone Sample Mill, Fort Collins, CO, USA) with a 0.5 mm screen. The alcoholic extracts of sorghum brans were obtained using aqueous ethanol solution (70% *v*/*v*) as described previously [17]. Briefly, one gram of each sample was combined with 9 mL 70% *v*/*v* ethanol. Samples were then briefly vortexed before being placed on a shaker at room temperature for 2 h. After shaking, samples were placed at −20 °C overnight. On the next day, samples were then centrifuged at 4 °C for 10 min at 2970× *g* [19]. Supernatants were collected for chemical analysis and use in tissue culture experiments.

### 2.3. Total Phenolic Content (TPC) Assay

The TPC assay was carried out using the Folin–Ciocalteu (FC) reagent assay [20] with adaptations [19,21]. Briefly, a standard curve of gallic acid (0–200 µg/L, R^2^ = 0.99) was used to estimate the TPC in terms of gallic acid equivalents (GAE). The FC reagent was diluted in deionized water at 1:1 (*v*/*v*) working ratio. Liquid extracts were diluted in 70% ethanol to achieve acceptable absorbance values. After sample dilution, 25 µL of both standards and samples were added to a 96-well plate in triplicate and combined with 75 µL of deionized water and 25 µL of working FC reagent using a 96-channel semi-automated pipettor (Sorenson Bioscience Inc., Radnor, PA, USA). After a 6 min waiting period, 100 µL of 7.5% Na_2_CO_3_ was added, and a heat-sealing film (Eppendorf North America, Inc., Enfield, CT, USA) was immediately applied to seal the plate before being placed in the dark for 90 min. After the incubation period, sample absorbance was measured at 765 nm using a Biotek H4 plate reader (Biotek, Winooski, VT, USA). The TPC results of triplicates were expressed in terms of in milligram GAE per gram raw material.

### 2.4. Vanillin Assay for Condensed Tannin Quantification

The vanillin assay was performed as described previously [22]. Briefly, all solutions were prepared in 100% methanol; 1% vanillin (1 g in 100 mL), catechin (10 mg in 10 mL), 4% HCl (*v*/*v*)), 8% HCl (*v*/*v*). Working vanillin was prepared by mixing equal portions of 1% vanillin and 8% HCl-methanol. To remove the aqueous solvent, 1 mL of extract was dried for 5.5 h at 60 °C using a Vacufuge Plus (Eppendorf, Enfield, CT, USA). Then, 1 mL 1% HCl-methanol was added to the dried samples and allowed to stir for 20 min. Samples were then centrifuged at 805× *g* for 6 min and supernatant was used to measure condensed tannins. In a 96-well plate, 30 µL of sample was dispensed, followed by 150 µL of working vanillin reagent. Then, 150 µL of 4% HCl-methanol was added to each corresponding control well. Samples and standards were incubated for 20 min at 30 °C. After incubation, sample absorbance was read at 500 nm using a Synergy 2 microplate reader (Biotek Instruments Inc., Winooski, VT, USA). The catechin standard curve was developed using a concentration range of 0–5.0 mg/mL. Net absorbance of standards and samples were calculated by subtracting the average of the control wells from the corresponding sample or standard wells. Net absorbances of standards were plotted against concentration to plot a linear equation to extrapolate catechin equivalents (CE) of samples. Samples were expressed as mg CE per g of bran. Each sample was extracted and measured three separate times.

### 2.5. HPLC Analysis of Sorghum Extracts

An HPLC system coupled to a diode array detector (HPLC-DAD, 1260 Infinity Series, Agilent, Santa Clara, CA, USA) and equipped with a C18 column (Kinetex^®^ 5 µm EVO C18 100 Å, 150 mm × 4.6 mm, Phenomenex, Torrance, CA, USA) coupled with a XPERTEK^®^ pre-column inline filter (0.5 µm, P.J. Cobert, St. Louis, MO, USA) was used for the analysis. The solvents used in the mobile phase were (A) acetonitrile, (B) methanol, and (C) acidified water (0.5% acetic acid *v*/*v*). Samples and analytical standards solubilized in methanol:ethanol (90:10, *v*/*v*) were injected (20 µL) into HPLC with the following elution gradient at 1 mL/min and at a column temperature of 30 °C: the initial composition of the mobile phase A:B:C = 5:5:90 was changed with a linear gradient to 8:8:84 in 0–5 min, 10:10:80 in 5–15 min, 25:0:75 15–25 min, 30:0:70 25–35 min, and 60:0:40 35–45 min. A post time of 5 min was used.

The chromatograms were recorded at 260, 280, 320, 360, and 430 nm. For quantification of luteolinidin, eriodictyol, luteolin, naringenin, and apigenin, 280 nm was used, whereas for caffeic-, p-coumaric-, and ferulic acids, 7-methoxy-apigenidin, apigenidin, and kaempferol were quantified at 320 nm.

The alcoholic sorghum extract obtained from 1g of sorghum bran was dried at 60 °C, using a Rocket Synergy 2 Evaporation System (Thermo Fisher, Pittsburgh, PA, USA), and re-constituted (100 mg/10 mL) in acidified methanol (HCl 1% *v*/*v*) by stirring in a Ultrasonik Ney ultrasonic bath (Simi Valley, CA, USA), model for 10 min, and followed by centrifugation at 2 °C at 3500× *g*, time, Thermo Fisher, Pittsburgh, PA, USA) for 10 min. The insoluble pellet of the crude extract was discarded. The solvent was removed by flushing nitrogen (99% pure, Matteson, Manhattan, KS, USA). The sample was solubilized in 1 mL methanol: ethanol (90:10, *v*/*v*), filtered with a 0.45 µm polyvinylidene difluoride membrane (PVDF, Millipore, Lenexa, KS, USA), and injected into the HPLC system at 20 µL.

The selection of phenolic standards was based on phenolics previously found to be present in sorghum through a literature search [23,24,25,26,27,28,29]. Stock solutions of phenolic standards (1 mg/mL) were prepared in ethanol and the working solutions for the calibration curves (2.00 × 10^−5^–0.09 mg/mL, R^2^ ≥ 0.99) were prepared by diluting the stock solutions (dilution ratios of 1:11, 1:22, 1:50, 1:500, and 1:5000, *v*/*v*). Standard addition was used to accurately identify each compound. This method was modified based on the work of Iraki et al., 2012 [30].

Each extract was purified and measured four separate times. The results were expressed as µg target compound per g of bran.

### 2.6. Cell Culture, Differentiation of 3T3-L1 and Treatment of Sorghum Bran Extracts

3T3-L1 preadipocytes was cultured with DMEM containing 10% FBS until confluent. Differentiation of 3T3-L1 was performed as we described previously [31,32]. Two days after reaching confluence (day 0), cells were stimulated to differentiate by using a differentiation cocktail containing (DMI) 1 μM dexamethasone, 0.5 mM isobutylmethylxanthine, and 5 μg/mL insulin in 10% FBS/DMEM for 2 days (day 2). Subsequently, cells were maintained in 10% FBS/DMEM medium with 5 μg/mL insulin for another 2 days (day 4), followed by culturing with 10% FBS/DMEM medium until analysis. Different types of sorghum bran extracts were treated with cell culture media at concentrations indicated in the figures and figure legends.

### 2.7. Oil Red O (ORO) Staining

ORO staining of triglycerides and lipids was performed at the times indicated in the figure legend following the initiation of differentiation as described previously [32]. In brief, the cells were fixed with 10% formalin/phostate buffered saline and stained with 60% ORO working solution for 10 min. After washing, stained ORO was dissolved in isopropanol and the absorbance at 500 nm wavelength was measured using microplate reader (Bio-Tek Instruments Inc., Winooski, VT, USA).

### 2.8. Cell Viability

Viability of undifferentiated and differentiated 3T3-L1 cells was measured using the MTT assay as we described previously [33].

### 2.9. Reactive Oxygen Species (ROS) Production

Cellular ROS level was measured using fluorescent probe, 2′,7′-dichlorodihydrofluorescin diacetate (DCFH-DA) (Sigma-Aldrich, St Saint Louis, MO, USA ). On Day 10, cells were incubated with 50 μM of DCFH-DA in serum-free DMEM for 1 h. After washing, fluorescent intensity (Ex 460/Em 540) was measured using microplate reader (Bio-Tek Instruments Inc., Winooski, VT, USA).

### 2.10. Glucose Uptake

On day 10, differentiated 3T3-L1 cells were glucose starved in glucose-free medium (Gibco, #A1443001, Grand Island, NY, USA) for 2 h. After washing, cells were incubated with 50 μM of 2-(N-(7-Nitrobenz-2-oxa-1,3-diazol-4-yl)Amino)-2-Deoxyglucose (2-NBDG) (Invitrogen, CA, USA) in glucose-free medium for 1 h. Cells were washed with PBS twice and fluorescence intensity (Ex 460/Em 540) was measured using a microplate reader (Bio-Tek Instruments Inc., Winooski, VT, USA).

### 2.11. Lipolysis

Fully differentiated 3T3-L1 adipocytes were serum starved for 2 h and then treated with sorghum bran extracts for 24 and 48 h. Culture media was collected to measure glycerol contents using a Glycerol Cell-based Assay Kit (#10011725, Cayman Chemical, MI, USA) according to the manufacturer’s instructions. Briefly, 25 μL of culture media was mixed with 100 μL of glycerol detection reagent at room temperature for 15 min and then the absorbance at 540 nm was measured using a microplate reader (Bio-Tek Instruments Inc., Winooski, VT, USA).

### 2.12. Western Blot

The Western blot was performed as we described previously [33].

### 2.13. Statistical Analysis

Data are expressed as means ± SD as indicated in figure legends, and differences were considered significant at *p* < 0.05. Statistical analysis was performed with student’s *t*-test.

## 3. Results

### 3.1. Chemical Analysis of Sorghum Bran Extracts

**Figure 1:**
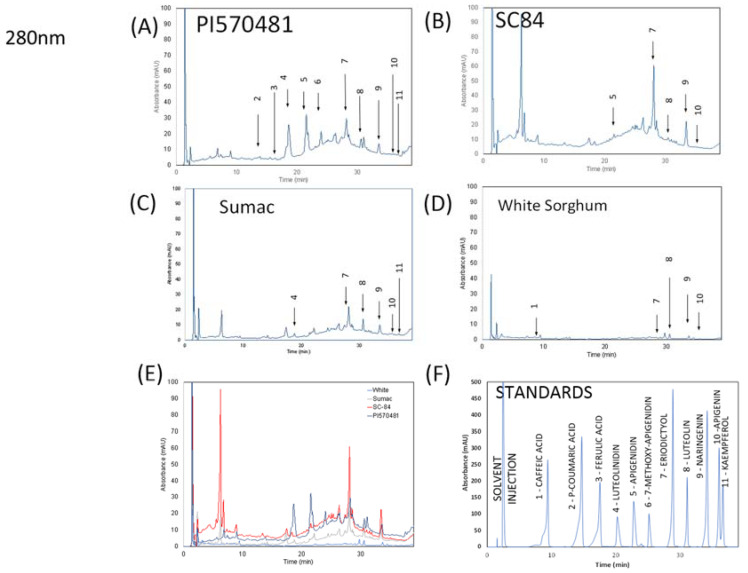

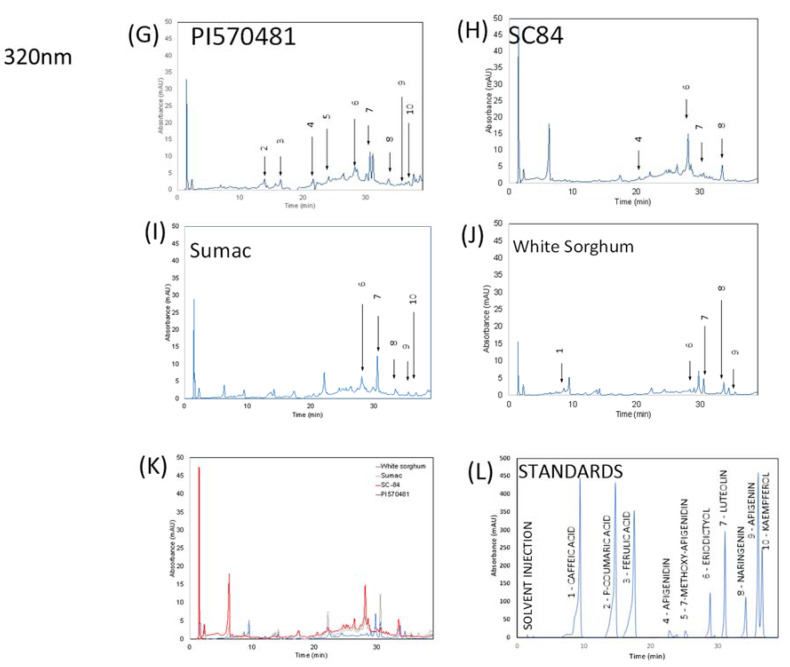
HPLC analysis of sorghum extracts. The chromatograms of sorghum varieties (5 mg/mL of dried extract) at 280 nm (**A**–**D**), sorghum genotype chromatograms overlaid at 280 nm (**E**) and the mixture of standards (0.09 mg/mL) at 280 nm (**F**), (5 mg/mL of dried extract) at 320 nm (**G**–**J**), sorghum genotype chromatograms overlaid at 320 nm (**K**), and the mixture of standards (0.09 mg/mL) at 320 nm (**L**).

Eleven phenolic compounds were identified in the sorghum extracts via HPLC-DAD (Table 1 and Figure 1). PI570481 extract was the only extract that contained p-coumaric acid (50.23 µg/g) and ferulic acid (15.70 µg/g). PI570481 contained the highest amounts of luteolinidin (607.61 µg/g), apigenidin (103.45 µg/g), 7-methoxy apigenidin (226.74 µg/g), luteolin (128.06 µg/g), and kaempferol (35.36 µg/g), when compared to the other extracts analyzed. PI570481 also contained eriodictyol (148.42 µg/g) and naringenin (32.74 µg/g). SC84 contained the highest amounts of eriodictyol (483.77 µg/g), when compared to the other three extracts analyzed. SC84 contained apigenidin (82.96 µg/g) and luteolin (68.78 µg/g). Sumac sorghum bran extract was the only extract to contain measurable Apigenin (32.52 µg/g). Sumac sorghum bran extract was determined to contain luteolinidin (22.15 µg/g), eriodictyol (38.98 µg/g), luteolin (57.01 µg/g), naringenin (15.32 µg/g), and kaempferol (12.52 µg/g). White sorghum bran extract was the only extract that contained caffeic acid (9.84 µg/g). White sorghum bran extract also contained luteolin (16.15 µg/g) and naringenin (1.94 µg/g).

Differences in the TPC were found in the genotypes analyzed (Table 1).

The total phenolic content (TPC) values detected in Sumac and white sorghum extracts were 12.4 and 0.67 mg GAE/g, respectively, while the novel high-polyphenol sorghum extracts had TPC values of 38.11 mg GAE/g for PI570481 and 55.57 mg GAE/g for SC84.

Condensed tannins were not detected in white sorghum bran extract while PI570481, SC84 and Sumac extracts contained 179.01, 415.83, and 13.03 mg CE/g, respectively. It is important to note that sorghum tannins and catechin standards had different reaction rates using the vanillin assay [34]. This indicates that the results obtained using the vanillin assay are a poor measure for absolute quantification of tannins but that it is still a valid method to relatively quantify tannin content when comparing between sorghum genotypes.

### 3.2. Sorghum Extracts Do Not Cause Cytotoxicity in 3T3-L1 Cells

**Figure 2:**
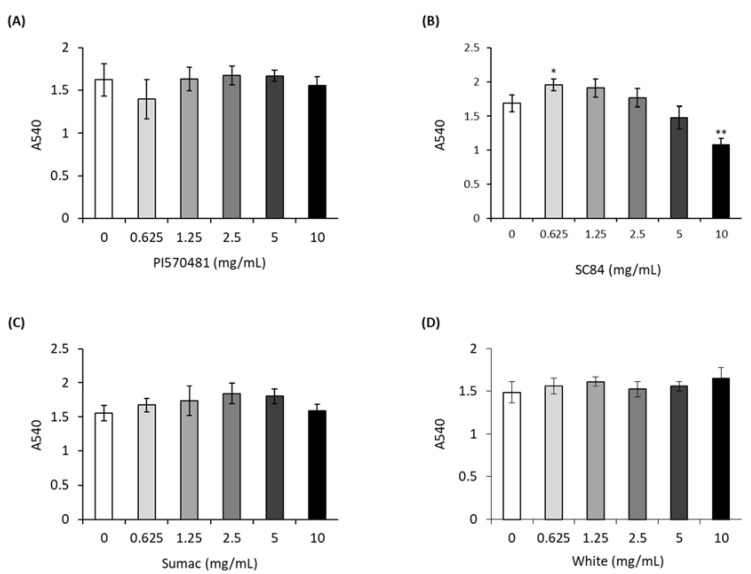
Sorghum bran extracts did not affect viability of 3T3-L1 cells. 3T3-L1 cells were treated with different doses of sorghum extracts ((**A**) PI570481, (**B**) SC84, (**C**) Sumac, (**D**) white) for 24 h and MTT assay was performed to measure the cytotoxicity. Values are means ± SD (*n* = 3). Significance is indicated as * *p* < 0.05, ** *p* < 0.01. Difference is between vehicle (0 mg/mL-treated) and treatment group.

According to recent studies, high-phenolic sorghum bran extracts suppressed viability of several types of colorectal and liver cancer cells [16,17]. To examine if sorghum bran extracts influence the viability of 3T3-L1 preadipocytes, we performed an MTT assay in undifferentiated fibroblast-like 3T3-L1 cells treated with different doses of sorghum bran extracts for 24 h. The results indicate that none of sorghum bran extracts caused changes in cell viability (Figure 2).

In addition, long term treatment of sorghum bran extracts (0, 0.625, 1.25, 2.5, and 5 mg/mL) for 10 days did not affect cell viability (Supplementary Figure S1).

### 3.3. Sorghum Bran Extracts Reduced Intracellular Lipid Accumulation in Differentiated 3T3-L1 Cells

**Figure 3:**
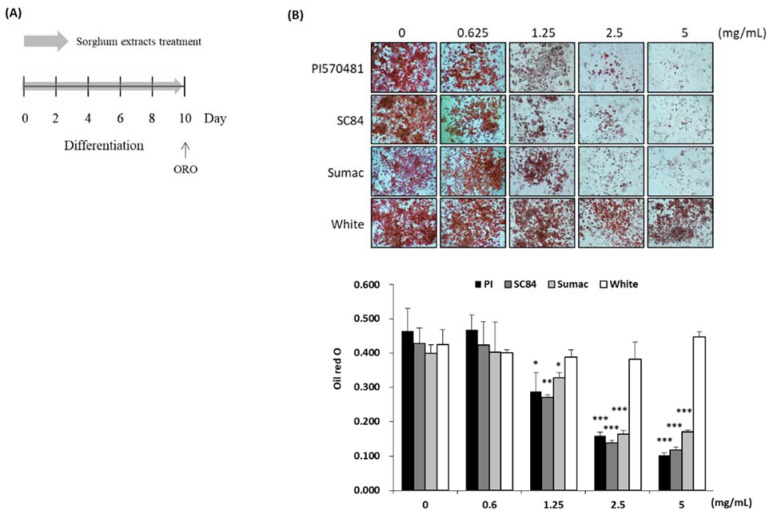
Sorghum bran extracts reduced intracellular lipid accumulation in differentiated 3T3-L1 cells. (**A**) Experimental design. (**B**) 3T3-L1 cells were differentiated with the DMI cocktail for 2 days and treated with maintenance media containing different concentrations of sorghum extracts (PI570481, SC84, Sumac, white) for 8 days (total 10 days). Intracellular lipid was measured by oil red O (ORO) staining and shown as an image. The ORO was dissolved in isopropanol and absorbance was measured at 500 nm and expressed as a graph. Values are means ± SD (*n* = 3). Significance is indicated as * *p* < 0.05, ** *p* < 0.01, *** *p* < 0.001. Difference is between vehicle (0 mg/mL-treated) and treatment group.

Adipogenesis (adipocyte differentiation) is the process of cell differentiation by which preadipocytes are converted to adipocytes. During this cellular process, the preadipocytes undergo cessation of proliferation and undergo morphologic changes that are characterized by lipid droplet accumulation, an indication of fully differentiated mature adipocytes [35]. We used the differentiation of the mouse 3T3-L1 preadipocyte cell line, which is the most used model system for adipogenesis studies. Different concentrations of sorghum bran extracts were used during the differentiation of 3T3-L1 preadipocytes from day 0 (treating differentiation media) until day 10 and ORO staining was performed (Figure 3A). As a result, large amounts of lipid were accumulated in the untreated differentiated adipocytes while treatment of sorghum bran extracts (PI570481, SC84, and Sumac) significantly suppressed lipid accumulation in a dose-dependent manner. The significance was observed from the cells treated with 1.25, 2.5, and 5 mg/mL sorghum extracts (37.9, 36.6, and 18.0% inhibition in 1.25 mg/mL; 65.7, 67.5, and 58.8% inhibition in 2.5 mg/mL; 78.2, 72.3, and 57.4% inhibition in 5.0 mg/mL of PI570481, SC84, and Sumac-treated cells, respectively). However, no change was observed in the cells treated with white sorghum extract, which was extracted from low-phenolic sorghum grain (negative control) (Figure 3B).

### 3.4. Sorghum Bran Extracts Repressed Expression of Adipogenic and Lipogenic Proteins in Differentiated 3T3-L1 Cells

**Figure 4:**
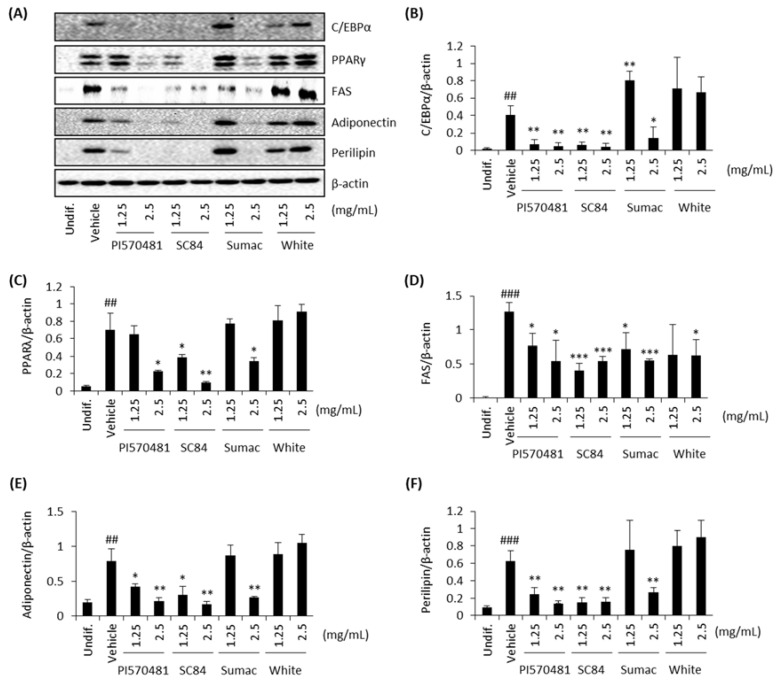
Sorghum bran extracts repressed expression of adipogenic and lipogenic proteins in differentiated 3T3-L1 cells. 3T3-L1 cells were treated with 0, 1.25, and 2.5 mg/mL sorghum extracts (PI570481, SC84, Sumac, white) for 10 days and Western blot was performed for C/EBPα, PPARγ, FAS, adiponectin, perilipin, and β-actin. (**A**) Representative western blot images (**B**–**F**) Densitometry data generated for Western blots. Values are means ± SD (*n* = 3). Significance is indicated as # and *. ## *p* < 0.01, ### *p* < 0.001 as compared to control (undifferentiated cells). * *p* < 0.05, ** *p* < 0.01, *** *p* < 0.001 as compared to untreated (0 mg/mL) differentiated cells.

A number of transcription factors, including C/EBPs (α, β, and δ) and PPARγ, have been identified as potential regulators of fat cell differentiation [36]. Since high-phenolic sorghum bran extract repressed adipocyte differentiation and lipid accumulation (Figure 4), we further examined the expression of genes that regulate adipocyte differentiation in vitro. Differentiation of 3T3-L1 cells with DMI cocktail dramatically stimulated the expression of adipogenic markers (CEBPα and PPARγ) and lipogenic marker proteins (fatty acid synthase, adiponectin, and perilipin). Treatment of sorghum bran extracts (PI570481, SC84, and Sumac) suppressed the differentiation-stimulated expression of adipogenic and lipogenic marker proteins significantly whereas no change was detected in white sorghum-treated cells (Figure 4).

### 3.5. Anti-Adipogenic Activities of Sorghum Bran Extracts Depend on Early Phase of Adipogenesis

**Figure 5:**
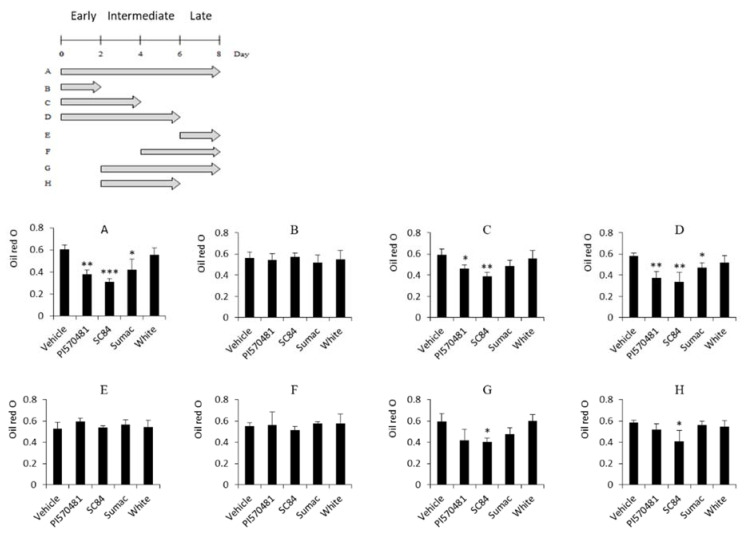
Anti-adipogenic activities of sorghum bran extracts depends on the early phase of adipogenesis. (**A**–**H**) ORO staining from differentiated 3T3-L1 cells treated with 2.5 mg/mL of sorghum extracts (PI570481, SC84, Sumac, white) for different times. Schematic diagram on the top shows time frame for treatment of sorghum bran extracts. Values are means ± SD (*n* = 3). Significance is indicated as * *p* < 0.05, ** *p* < 0.01, *** *p* < 0.001. Difference is between vehicle (0 mg/mL-treated) and treatment group. →: Incubation time with sorghum bran extracts.

Upon stimulating adipogenesis by DMI cocktail, over-confluent preadipocytes undergo the distinct stages of differentiation: the early, intermediate, and late stages with changes in the expression of cell-cycle regulators and genes related to adipogenesis [37]. To investigate which stage is critical for the sorghum bran-mediated suppression of adipogenesis, we treated sorghum extracts at different time points. As shown in Figure 5, similar suppression of intracellular lipid accumulation was observed in the differentiated cells treated from the early (day 0–2) to the intermediate (day 0–6) or late (day 0–8) stages (Figure 5A,C,D). However, none of cells treated with sorghum bran extracts after the early stage (day 0–2) showed anti-adipogenic activity (Figure 5E–H). In addition, the first half of the intermediate (day 2–4) stage is essential to see anti-adipogenic activity in sorghum bran extracts. These data indicate that sorghum bran extracts play a significant role in suppressing fat accumulation during the early stage of adipogenesis (day 0–4).

### 3.6. Sorghum Bran Extracts Repressed ROS Production and MAPK Signaling Pathways in Differentiated 3T3-L1 Cells

**Figure 6:**
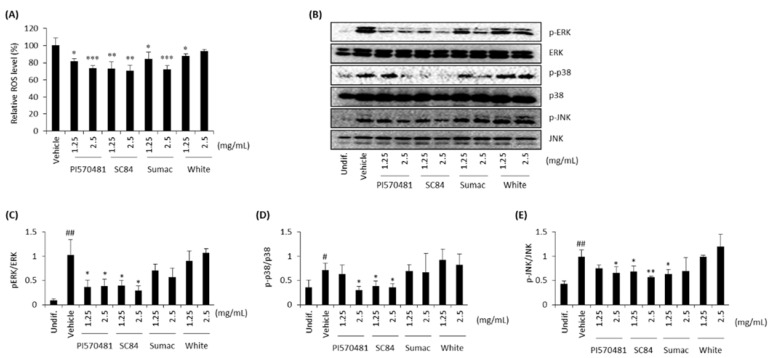
Sorghum bran extracts repressed ROS production and phosphorylation of MAPKs (ERK, p38, and JNK) in differentiated 3T3-L1 cells. (**A**) 3T3-L1 cells were treated with 0, 1.25, and 2.5 mg/mL sorghum extracts (PI570481, SC84, Sumac, white) for 10 days and ROS production was measured using DCFH-DA (*n* = 4). (**B**) Western blot was performed for phosphor- and total ERK, p38, and JNK (**B**–**E**). Values are means ± SD (*n* = 3). Significance is indicated as # and *. # *p* < 0.05, ## *p* < 0.01 as compared to control (undifferentiated cells). * *p* < 0.05, ** *p* < 0.01, *** *p* < 0.001 as compared to untreated (0 mg/mL) differentiated cells.

Reactive oxygen species (ROS) are generated during the differentiation of adipocytes, as well as by other cell metabolic processes, promoting adipogenesis. The effect of ROS on adipogenesis is known to be suppressed by antioxidants or ROS scavengers [38,39]. Therefore, we investigated if ROS are produced and released during adipogenesis and if the anti-adipogenic activity of sorghum bran extracts could be associated with reduced ROS production. The results indicate that ROS production was increased in differentiated cells while sorghum bran extracts reduced ROS production (Figure 6A). An intracellular signaling pathway type that plays a pivotal role in the adipocyte differentiation of preadipocyte-established cell lines is MAPKs, including ERK, p38, and JNK [40], which are major downstream target of the signaling of insulin, a major adipogenic inducer. In addition, increased ROS production also leads to the activation of MAPKs, including ERK, p38, and JNK [41]. Therefore, we tested if sorghum bran extracts alleviate insulin-stimulated MAPK pathways. Prominent induction of these three MAPKs in differentiated cells were observed while sorghum bran extracts reduced phosphorylation of three MAPKs (Figure 6B–E).

### 3.7. Sorghum Bran Extracts Repressed Insulin Signaling and Glucose Uptake in Differentiated 3T3-L1 Cells

**Figure 7:**
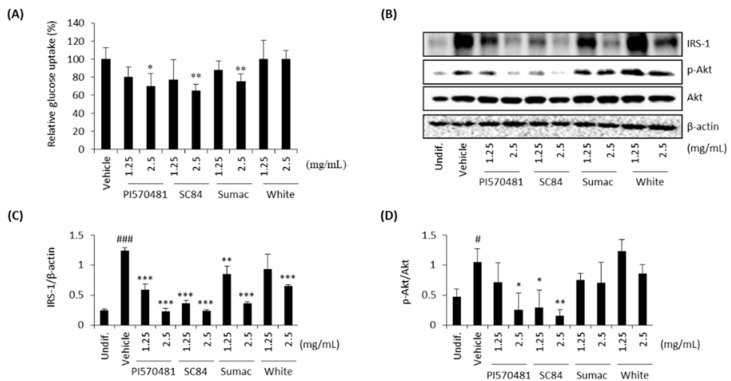
Sorghum bran extracts repressed glucose uptake and insulin signaling in differentiated 3T3-L1 cells. (**A**) 3T3-L1 cells were treated with 0, 1.25, and 2.5 mg/mL sorghum extracts (PI570481, SC84, Sumac, white) for 10 days. Glucose uptake was measured using 2-NBDG (*n* = 4). (**B**) Western blot was performed for IRS-1 and phospho and total Akt (**B**–**D**). Values are means ± SD (*n* = 3). Significance is indicated as # and *. # *p* < 0.05, ### *p* < 0.001 as compared to control (undifferentiated cells). * *p* < 0.05, ** *p* < 0.01, *** *p* < 0.001 as compared to untreated (0 mg/mL) differentiated cells.

Insulin regulates the development and function of adipose tissue and stimulates the differentiation program of adipose cells via an intracellular signaling cascade involving the insulin receptor (IR), insulin receptor substrate (IRS) proteins, phosphoinositol 3-kinase (PI3K), and protein kinase B (Akt) [42]. Insulin signaling requires glucose to promote lipid anabolism in adipocytes [43] and insulin-dependent glucose uptake is essential for adipocyte differentiation and maturation in the de novo synthesis of lipids (maturation) in developing adipocytes [44]. Therefore, we examined if sorghum bran extracts affect insulin signaling and glucose uptake. As shown in Figure 7A, glucose uptake was reduced in differentiated 3T3-L1 cells treated with sorghum bran extracts (PI570481, SC84, and Sumac), except for white sorghum F1000. We further measured IRS-1 and the phosphorylation of Akt, which are major components of insulin signals. Activation of these insulin signaling proteins was reduced by the treatment of sorghum bran extracts (PI570481, SC84, and Sumac), but not with white sorghum (Figure 7B–D).

### 3.8. Sorghum Bran Extracts Did Not Affect Lipolysis and Apoptosis of Differentiated in 3T3-L1 Cells

One of major hallmarks of lipid catabolism is lipolysis and subsequent fatty acid oxidation, which are inversely associated with intracellular fat accumulation in adipocytes. Thus, we investigated the lipolytic activity by measuring glycerol release and the expression of hormone sensitive lipase (HSL). Treatment with sorghum extracts for 24 h and 48 h did not induce glycerol release, although reduction was detected in the cells treated with 2.5 mg/mL SC84 and Sumac for 24 h (Supplementary Figure S2). Another significant mechanism of the anti-obesity effect is the induced apoptosis, which is a promising target to prevent obesity. Since many anti-obesity properties of phenolic compounds are mediated by the induction of apoptosis in fully differentiated adipocytes [45,46], we also examined whether sorghum bran extract increased apoptosis in differentiated adipocytes. The results indicate that no change in the expression of the molecular marker (PARP cleavage) and pro-apoptotic proteins (Bak and Bim) was observed in the cells treated with sorghum extracts (Supplementary Figure S3).

## 4. Discussion

Health benefits of sorghum have been studied by many research groups focusing on the prevention of human chronic diseases including obesity [6]. The obesity-preventive activity of diverse sorghum extracts was reported [7,8,9,10,11,12,13,14].

Obesity is the consequence of an energy imbalance and is controlled by the lipid metabolism. The net lipid balance is determined by energy storage and energy combustion. To provide a reliable mechanism, we investigated the effects of high-phenolic sorghum bran extracts on lipid anabolic events (adipogenesis and lipogenesis) and energy catabolic events (lipolysis and fatty acid oxidation) including apoptosis. We hypothesize that reduced intracellular lipid accumulation is associated with the modulation of the proposed cellular events of lipid metabolism in adipocytes. Here, we report that sorghum bran extracts repress adipogenesis and lipogenesis without changing in catabolic pathways such as lipolysis and apoptosis.

Adipogenesis (fat cell differentiation) is a long-lasting and complex process accompanied with up- and down-regulation of many genes, controlling the cell cycles, transcriptional switches, and induction of many lipogenic genes. We found that sorghum bran extracts repressed the adipogenic process by interfering at both the early stage (day 0–2) and the first half of the intermediate stage (day 2–4). These are the time points for the overexpression of adipogenic regulators such as C/EBPα,β,γ and PPARγ and this implies that the lipid-lowering effect of sorghum extracts is adipogenic transcription factor-dependent. These results could also be another reason why lipolysis at the late stage of adipogenesis was not affected by treatment of sorghum bran extracts (Supplementary Figure S3).

Among many proposed adipogenic mechanisms, ROS facilitate adipocyte differentiation by accelerating mitotic clonal expansion [39]. Since sorghum bran extracts contain high amounts of anti-oxidative polyphenolic compounds, we hypothesized that reduced ROS by sorghum bran extracts could be reliable anti-adipogenesis mechanism. As shown in Figure 6A, ROS production was dramatically reduced by the treatment of sorghum bran extracts. In addition, activation of MAPK is a common response of insulin-stimulated adipogenesis. We also found that differentiation stimulated the phosphorylation of ERK, p38, and JNK, which was alleviated by treatment of sorghum extracts (Figure 6B–E). We believe that the suppression of ROS and MAPK activation are independent primary anti-adipogenic mechanisms. There exists a possibility that these two mechanisms are not mutually exclusive because increased ROS production also leads to the activation of MAPKs, including ERK, p38, and JNK [41].

It is important to note that the Sumac bran extract contained significantly fewer total polyphenols and condensed tannins when compared to PI570481 and SC84 but had a comparable anti-audiogenic effect as measured by Oil Red O staining. Similar effects were previously observed in a mouse colon cancer model, where Sumac had comparable efficacy to PI570481 and SC84 [47]. In the future, it will be important to study fractions, isolated tannins, and individual polyphenols in order to determine which components of the sorghum bran exert the anti-adipogenic effect. In particular, eriodictyol, luteolin, and naringenin, which were found in all three polyphenol-containing sorghum brans, should be analyzed for their anti-adipogenic effect. At the same time, analyzing fractions of the extract is of importance because there exists a possibly that components of the extract other than polyphenols exert the anti-adipogenic effect.

One of the interesting findings is that sorghum bran extracts inhibited insulin signaling and subsequent glucose uptake, which would be another mechanism explaining why sorghum bran extracts inhibited lipogenesis because glucose is dispensable for lipogenesis in adipocytes [43].

On the other hand, AMPK (AMP-activated protein kinase) plays a significant role in maintaining cellular energy homeostasis and is activated in response to a shortage of energy [48]. AMPK regulates fat cell homeostasis by regulating adipogenesis and lipolysis [49] and has been a target signal for many anti-obesity compounds [50]. However, our data indicate that AMPK is not mediator of the sorghum bran-mediated suppression of adipogenesis (data not shown). In the future, it will be important to study the effects of extracts obtained using other extraction methods because all the polyphenols are unlikely to be extracted using a single method.

**Figure 8:**
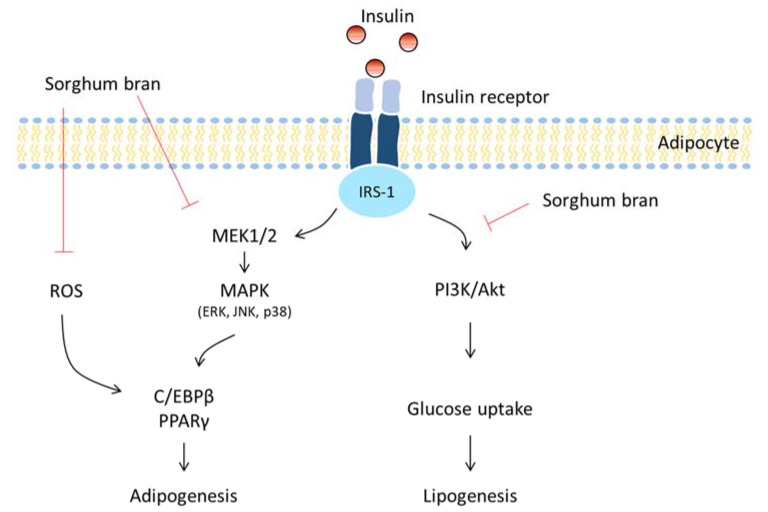
The schematic diagram proposing anti-adipogenic and anti-lipogenic mechanisms of sorghum bran extracts in differentiated 3T3-L1 cells.

In summary, sorghum bran extracts repressed fat accumulation in differentiated adipocytes without changes in cell viability. The results extend our knowledge on the mechanism by which sorghum bran consumption exerts anti-obesity effects. They include (1) the repression of ROS production and MAPK signaling, leading to the suppression of adipogenesis and (2) the repression of insulin signaling, leading to the suppression of glucose uptake and lipogenesis (Figure 8).

## Figures and Tables

**Table 1:** Identified polyphenol compounds, total phenolics, and condensed tannins in four sorghum genotypes.

Compound	Peak Number	Wavelength (nm)	Retention Time (min)	PI570481	SC84	Sumac	White
Caffeic acid	1	320	8.64 ± 0.05	ND†	ND†	ND†	9.84 ± 0.04
p-Coumaric acid	2	320	13.43 ± 0.07	50.23 ± 0.33	ND†	ND†	ND†
Ferulic acid	3	320	16.06 ± 0.08	15.70 ± 1.71	ND†	ND†	ND†
Luteolinidin	4	280	18.40 ± 0.50	607.61 ± 145.21	ND†	22.15 ± 3.20	ND†
Apigenidin	5	280	21.32 ± 0.40	103.45 ± 19	82.96 ± 8.37	ND†	ND†
7-methoxy apigenidin	6	320	24.03 ± 0.29	226.74 ± 20.72	ND†	ND†	ND†
Eriodictyol	7	280	28.23 ± 0.26	148.42 ± 95	483.77 ± 32.24	39.98 ± 2.40	NQ‡
Luteolin	8	280	30.69 ± 0.30	128.06 ± 4.35	68.78 ± 1.05	57.01 ± 0.48	16.15 ± 0.34
Naringenin	9	280	33.48 ± 0.05	32.74 ± 0.75	98.78 ± 2.48	15.32 ± 0.48	1.94 ± 0.13
Apigenin	10	320	35.55 ± 0.06	NQ‡	NQ‡	33.52 ± 0.05	NQ‡
Kaempferol	11	320	36.12 ± 0.13	35.36 ± 0.06	ND†	12.52 ± 0.03	ND†
Total polyphenols mg GAE/g *				38.11 ± 1.38	55.57 ± 4.78	12.4 ± 0.67	0.67 ± 0.88
Condensed Tannins mg CE/g ^#^				179.01 ± 20.99	415.83 ± 89.61	13.03 ± 0.27	ND

* Measured by Folin–Ciocalteu assay. ^#^ Measured by Vannillin assay. ND†—Non-detected, under the Limit of Detection and equipment used. NQ‡—Detected but not quantified. Limit of Quantification below 0.0002 mg/mL.

## Data Availability

All data are published within the manuscript. Original images and data can be obtained by contacting one of the corresponding authors.

## Supplementary Materials

The following supporting information can be downloaded at: https://www.mdpi.com/article/10.3390/nu14071493/s1, Figure S1: Long term cell Viability. 10-day cell viability assay of 3T3-L1 pre-adipocytes treated with (A) PI570481, (B) SC84, (C) Sumac, (D) White sorghum, Figure S2: Glycerol Release Glycerol release after (A) 24 and (B) 48 hrs of treatment with sorghum bran extracts, Figure S3: Western blot analysis of apoptotic proteins. (A) PARP, Bak, Bim with data quantified in (B) PARP, (C) Bak and (D) Bim (normalized to actin), Table S1: The area under curve of prominent unidentified peaks found in sorghum varieties at 280 nm.
