# Supplementary material for: Anti-Adipogenic Activity of High-Phenolic Sorghum Brans in Pre-Adipocytes
Source: Nutrients. 2022 Apr 2;14(7):1493. doi: 10.3390/nu14071493 (PMC9002988; doi:10.3390/nu14071493)
Supplement: Supplementary file 1 [file nutrients-14-01493-s001.zip › nutrients-1660676-supplementary.pdf]

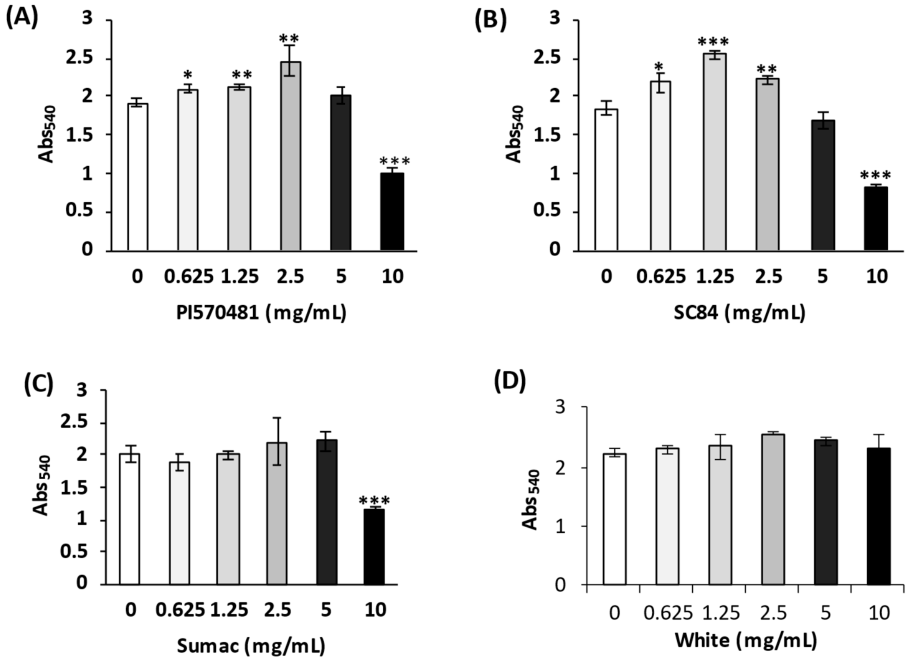


**Figure S1**. Long term cell Viability. 10-day cell viability assay of 3T3-L1 pre-adipocytes treated with (**A**) PI570481 ,(**B**) SC84 ,(**C**) Sumac , (**D**) White sorghum. *, $p$ < 0.05; **, $p$ < 0.01 ;***, $p$ < 0.001, when compared to control .

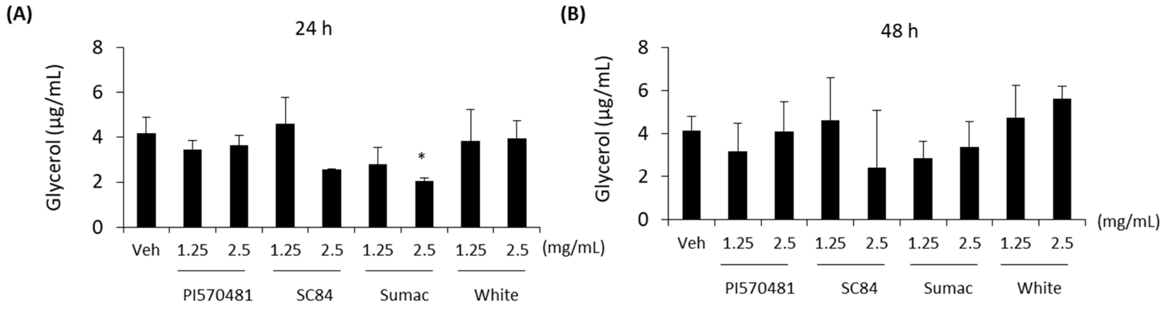


**Figure S2.** Glycerol Release Glycerol release after (**A**) 24 and (**B**) 48 hrs of treatment with sorghum bran extracts. *, $p < 0.05$, when compared to control.

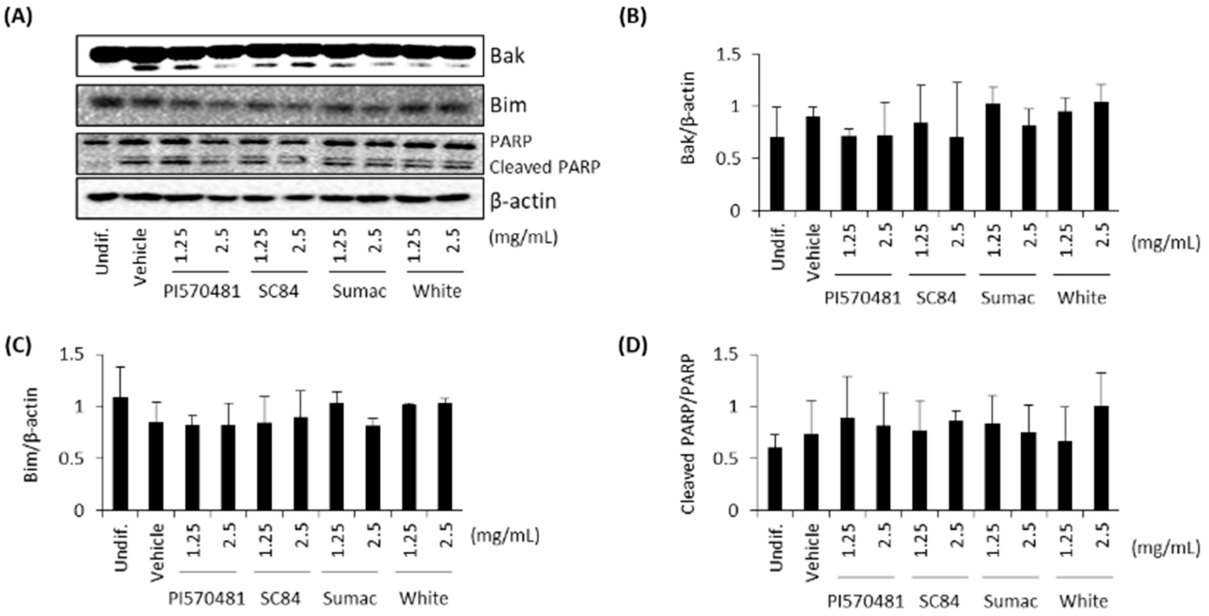


**Figure S3**: Western blot analysis of apoptotic proteins. (**A**) PARP, Bak, Bim with data quantified in (**B**) PARP, (**C**) Bak and (**D**) Bim (normalized to actin).

**Table S1**. The area under curve of prominent unidentified peaks found in sorghum varieties at 280 nm .

| | | | Area (mAU x s) | | | |
|---|---|---|---|---|---|---|
| **Peak number** | **Wavelenght (nm)** | **Retention time (min)** | **SC84** | **Sumac** | PI570481 | F1000 |
| 1 | 280 | 6.25 | 1288.56 | 211.36 | | |
| 2 | 280 | 6.76 | 181.53 | | | |
| 3 | 280 | 7.32 | 24.89 | | | |
| 4 | 280 | 9.01 | 90.99 | | 43.87 | |
| 5 | 280 | 26.34 | 134.22 | 88.38 | 73.71 | |
| 6 | 280 | 29.76 | | | | 59.37 |
| 7 | 280 | 31.08 | | | 123.29 | |
| 8 | 280 | 34.5 | | | | 16.57 |
| 9 | 280 | 39.15 | | | 135.41 | |
